# Supplementary material for: Effect of Sex on Clinical Outcomes in Patients with Coronavirus Disease: A Population-Based Study
Source: J Clin Med. 2020 Dec 24;10(1):38. doi: 10.3390/jcm10010038 (PMC7794723; doi:10.3390/jcm10010038)
Supplement: Supplementary file 1 [file jcm-10-00038-s001.pdf]

Supplementary Materials

Table S1. Abbreviations in the study.

Figure S1. Kaplan-Meier survival curves for populations aged 50–64 (A) or ≥65 (B) years.

Figure S2. Forest plot of the association between sex and mortality in patients according to the subgroups.

Table S2. Clinical outcomes according to sex for the different age groups.

Figure S3. Forest plot of the association between sex and clinical outcomes in patients according to age (A), DM (B), or HTN (C).

Figure S4. (A) Proportions of patients with diabetes mellitus or hypertension according to sex and age. (B) CCI scores according to sex and age.

Table 1. Abbreviations in the study.

ACE-2: angiotensin-converting enzyme-2
AHF: acute heart failure
AKI: acute kidney injury
CCI: Charlson comorbidity index
COVID-19: Coronavirus disease
DM: diabetes mellitus
ECMO: extracorporeal membrane oxygenation
HFNC: high flow nasal cannula
HIRA: Health Insurance Review and Assessment Service
ICD-10: International Classification of Diseases, 10th revision
MI: myocardial infarction
MV: mechanical ventilation
SARS-CoV-2: severe acute respiratory syndrome coronavirus 2
TMPRSSS2: transmembrane serine protease-2

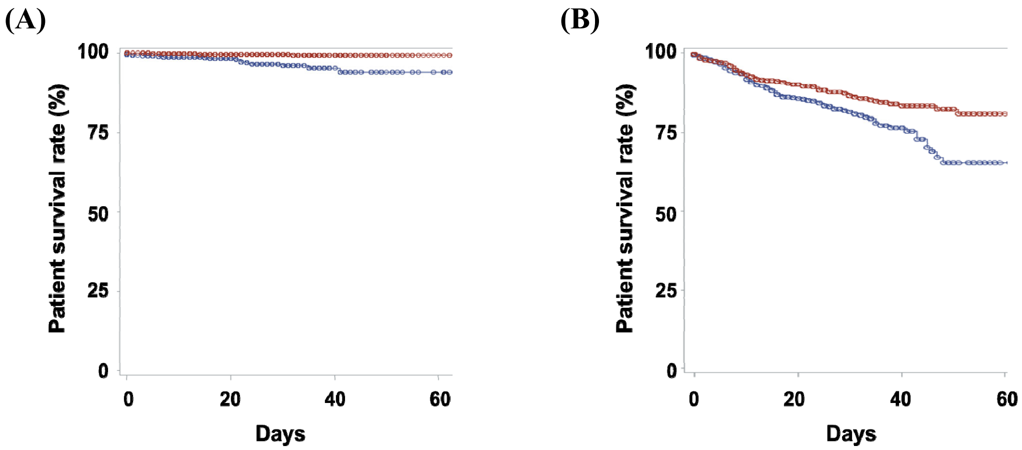

Figure S1. Kaplan-Meier survival curves for populations aged 50–64 (A) or ≥65 (B) years. The blue and red lines show the survival graphs for men and women, respectively. The circle presents the censored point. (A) The 20-day survival rates were 98.3% and 99.7% for men and women, respectively. The 40-day survival rates were 95.5% and 99.7% for men and women, respectively. (B) The 20-day survival rates were 85.6% and 90.1% for men and women, respectively. The 40-day survival rates were 76.3% and 83.2% for men and women, respectively.

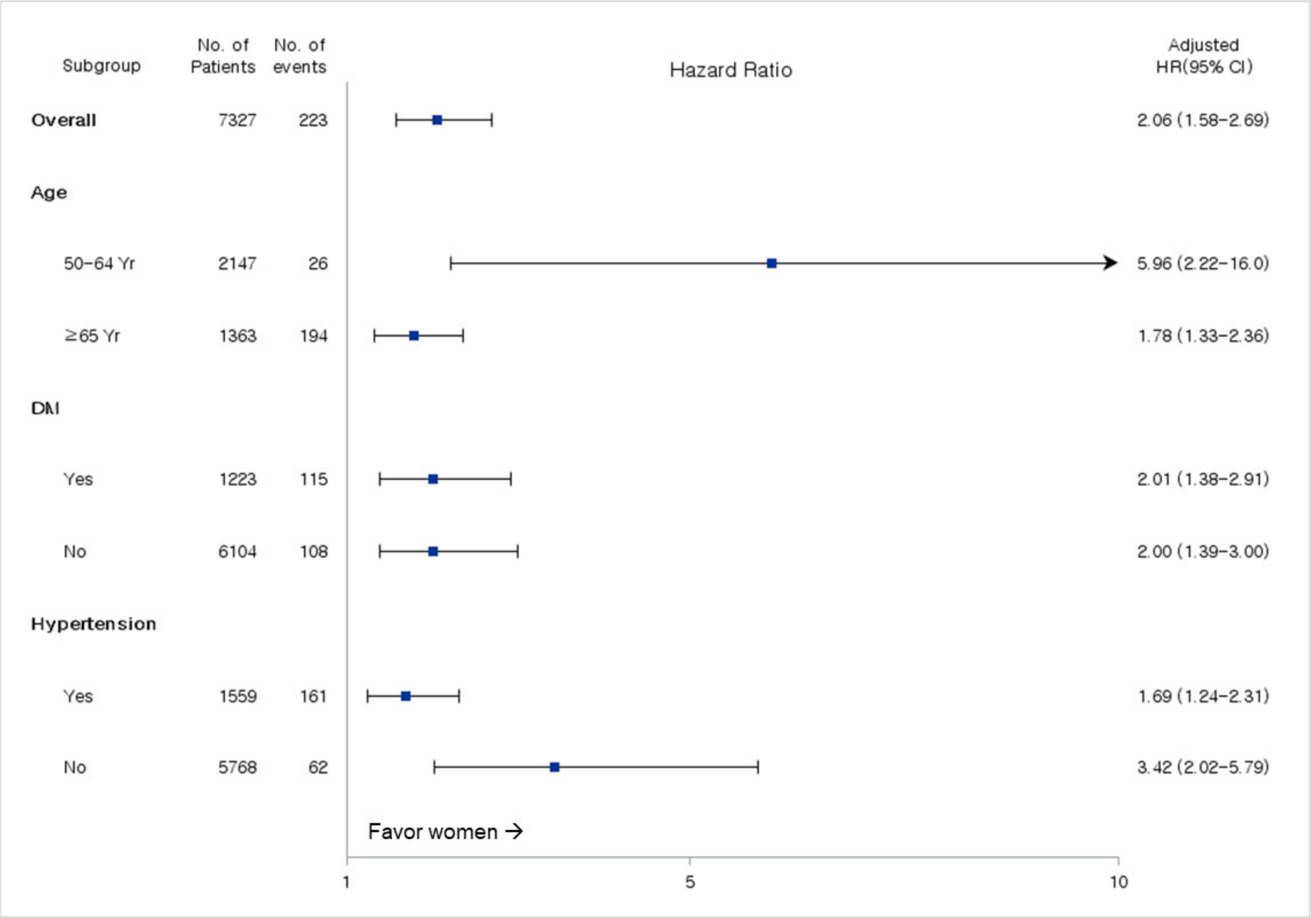

Figure S2. Forest plot of the association between sex and mortality in patients according to the subgroups. Multivariate analysis was adjusted for age, sex, CCI score, and hypertension. Multivariate analyses for hypertension subgroups were adjusted for age, sex, and CCI score. Abbreviations: DM, diabetes mellitus; HR, hazard ratio; CI, confidence interval; CCI, Charlson comorbidity index.

Table S2. Clinical outcomes according to sex for the different age groups.

	Total	Men	Women	*P*-value
**< 35 aged**				
Acute kidney injury	0	0	0	-
Inotropics	1 (0.04%)	1 (0.1%)	0	0.469
Conventional oxygen therapy	31 (1.3%)	19 (1.7%)	12 (0.9%)	0.106
High flow nasal cannula	1 (0.04%)	0	1 (0.1%)	1.000
Mechanical ventilation	0	0	0	-
Extracorporeal membrane oxygenation	0	0	0	-
Cardiac arrest	0	0	0	-
Myocardial infarction	81 (3.4%)	37 (3.3%)	44 (3.4%)	0.821
Acute heart failure	46 (1.9%)	24 (2.1%)	22 (1.7%)	0.471
**35-49 aged**				
Acute kidney injury	3 (0.2%)	3 (0.6%)	0	0.041
Inotropics	6 (0.4%)	5 (1.0%)	1 (0.1%)	0.021
Conventional oxygen therapy	67 (4.8%)	40 (8.2%)	27 (2.9%)	<0.001
High flow nasal cannula	7 (0.5%)	5 (1.0%)	2 (0.2%)	0.053
Mechanical ventilation	4 (0.3%)	4 (0.8%)	0	0.014
Extracorporeal membrane oxygenation	1 (0.1%)	1 (0.2%)	0	0.347
Cardiac arrest	1 (0.2%)	1 (0.2%)	0	0.347
Myocardial infarction	31 (2.2%)	14 (2.9%)	17 (1.9%)	0.252
Acute heart failure	64 (4.6%)	17 (3.5%)	47 (5.1%)	0.163

**50-64 aged**				
Acute kidney injury	6 (0.3%)	5 (0.6%)	1 (0.1%)	0.028
Inotropics	49 (2.3%)	32 (4.0%)	17 (1.3%)	<0.001
Conventional oxygen therapy	281 (13.1%)	139 (17.5%)	142 (10.5%)	<0.001
High flow nasal cannula	44 (2.0%)	29 (3.7%)	15 (1.1%)	<0.001
Mechanical ventilation	37 (1.7%)	24 (3.0%)	13 (1.0%)	<0.001
Extracorporeal membrane oxygenation	11 (0.5%)	7 (0.9%)	4 (0.3%)	0.112
Cardiac arrest	10 (0.5%)	8 (1.0%)	2 (0.1%)	0.007
Myocardial infarction	78 (3.6%)	36 (4.5%)	42 (3.1%)	0.086
Acute heart failure	109 (5.1%)	51 (6.4%)	58 (4.3%)	0.029
**≥65 aged**				
Acute kidney injury	18 (1.3%)	9 (1.6%)	9 (1.1%)	0.408
Inotropics	126 (9.2%)	62 (11.2%)	64 (7.9%)	0.037
Conventional oxygen therapy	522 (38.3%)	231 (41.8%)	291 (28.2%)	0.026
High flow nasal cannula	126 (9.2%)	67 (12.1%)	59 (7.3%)	0.002
Mechanical ventilation	81 (5.9%)	43 (7.8%)	39 (4.8%)	0.023
Extracorporeal membrane oxygenation	8 (0.6%)	3 (0.5%)	5 (0.6%)	1.000
Cardiac arrest	31 (2.3%)	20 (3.6%)	11 (1.4%)	0.006
Myocardial infarction	66 (4.8%)	35 (6.3%)	31 (3.8%)	0.034

Acute heart failure	161 (11.8%)	65 (11.8%)	96 (11.8%)	0.972

The data are expressed as number (percentage).

(A)

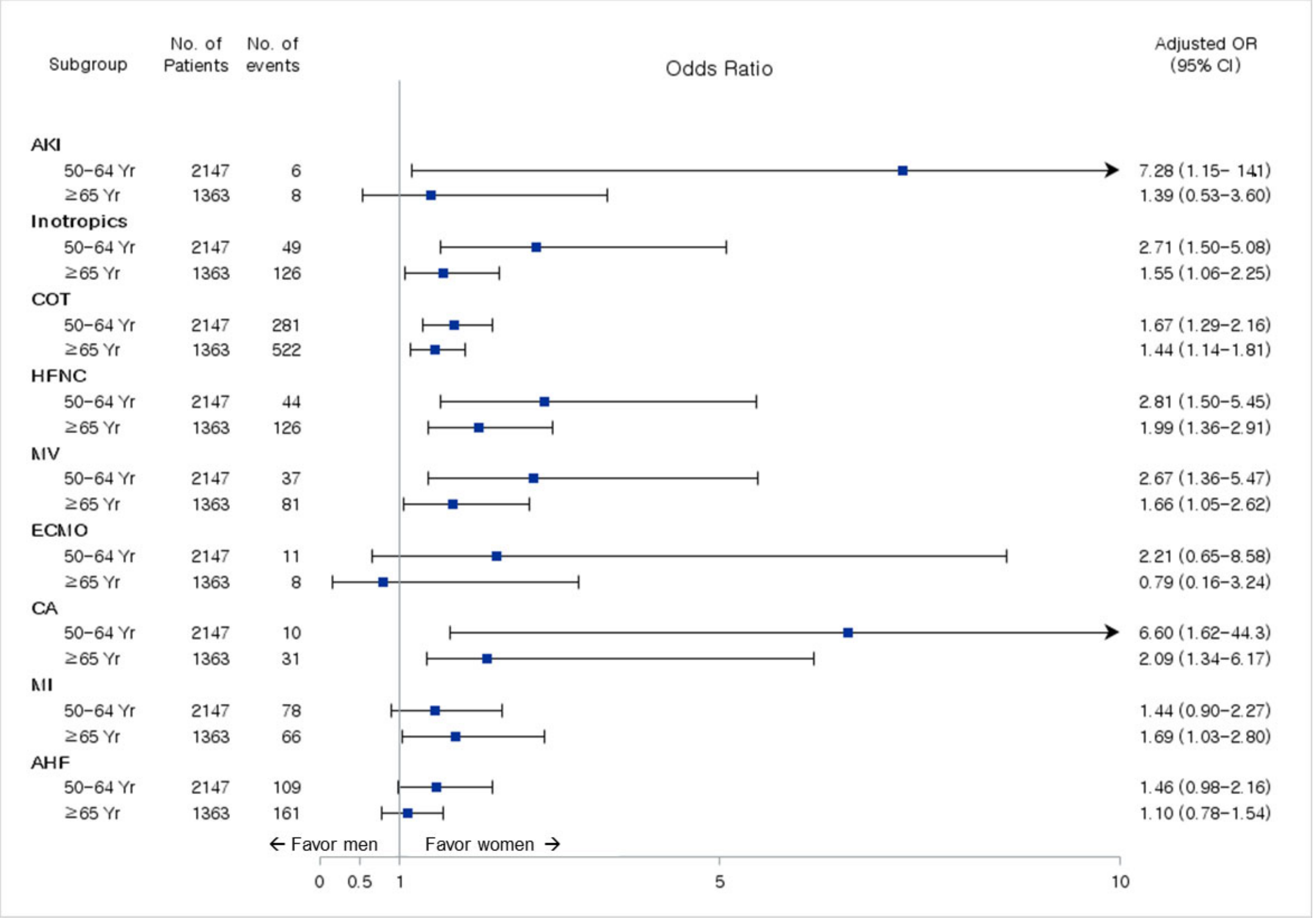
Subgroup
No. of Patients
No. of events
Odds Ratio
Adjusted OR (95% CI)
AKI
50–64 Yr 2147 6 7.28 (1.15– 141)
≥65 Yr 1363 8 1.39 (0.53–3.60)
Inotropics
50–64 Yr 2147 49 2.71 (1.50–5.08)
≥65 Yr 1363 126 1.55 (1.06–2.25)
COT
50–64 Yr 2147 281 1.67 (1.29–2.16)
≥65 Yr 1363 522 1.44 (1.14–1.81)
HFNC
50–64 Yr 2147 44 2.81 (1.50–5.45)
≥65 Yr 1363 126 1.99 (1.36–2.91)
MV
50–64 Yr 2147 37 2.67 (1.36–5.47)
≥65 Yr 1363 81 1.66 (1.05–2.62)
ECMO
50–64 Yr 2147 11 2.21 (0.65–8.58)
≥65 Yr 1363 8 0.79 (0.16–3.24)
CA
50–64 Yr 2147 10 6.60 (1.62–44.3)
≥65 Yr 1363 31 2.09 (1.34–6.17)
MI
50–64 Yr 2147 78 1.44 (0.90–2.27)
≥65 Yr 1363 66 1.69 (1.03–2.80)
AHF
50–64 Yr 2147 109 1.46 (0.98–2.16)
≥65 Yr 1363 161 1.10 (0.78–1.54)
← Favor men
Favor women →
0
0.5
1
5
10

(B)

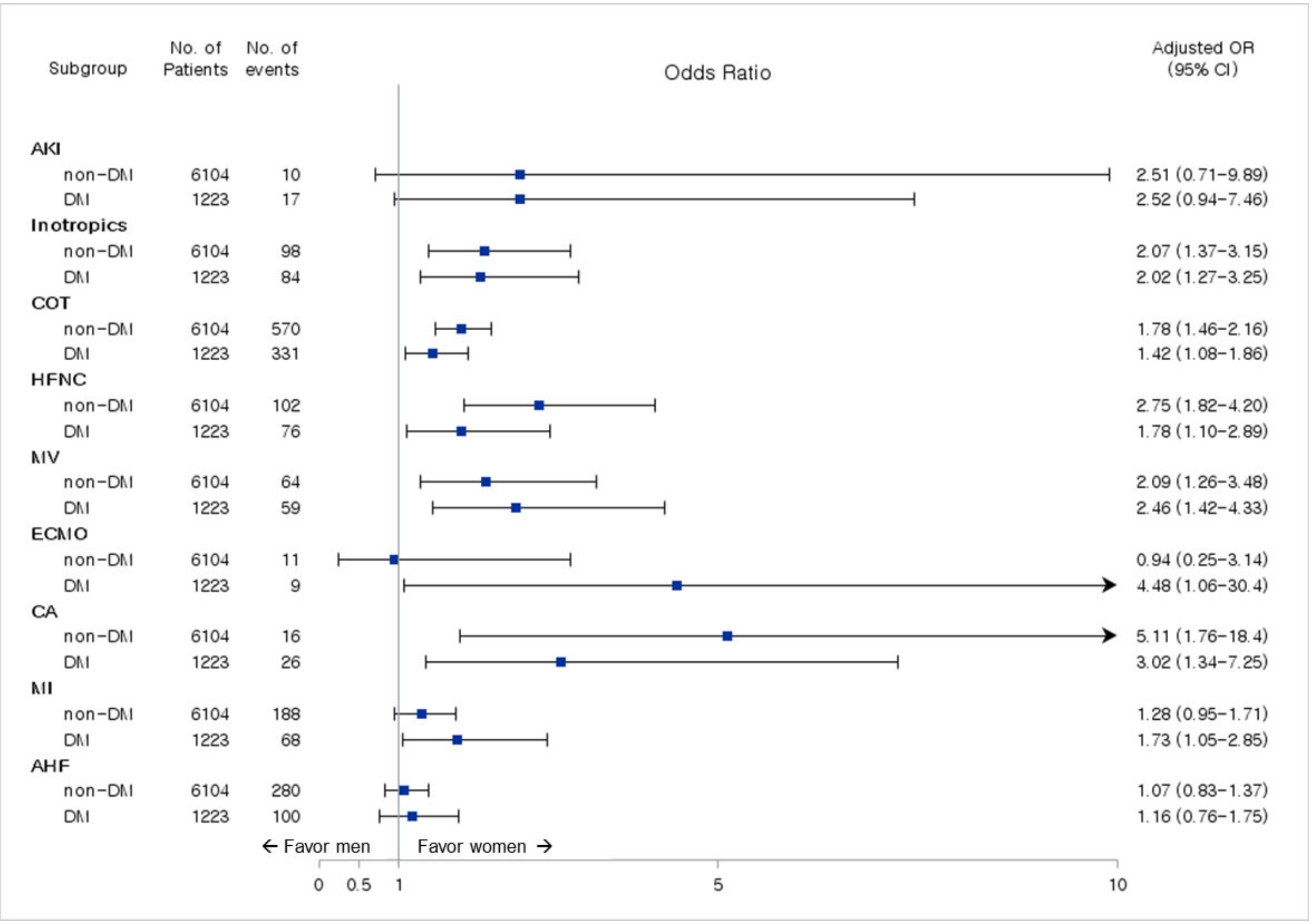
Subgroup
No. of Patients
No. of events
Odds Ratio
Adjusted OR (95% CI)
AKI
non-DM 6104 10 2.51 (0.71-9.89)
DM 1223 17 2.52 (0.94-7.46)
Inotropics
non-DM 6104 98 2.07 (1.37-3.15)
DM 1223 84 2.02 (1.27-3.25)
COT
non-DM 6104 570 1.78 (1.46-2.16)
DM 1223 331 1.42 (1.08-1.86)
HFNC
non-DM 6104 102 2.75 (1.82-4.20)
DM 1223 76 1.78 (1.10-2.89)
MV
non-DM 6104 64 2.09 (1.26-3.48)
DM 1223 59 2.46 (1.42-4.33)
ECMO
non-DM 6104 11 0.94 (0.25-3.14)
DM 1223 9 4.48 (1.06-30.4)
CA
non-DM 6104 16 5.11 (1.76-18.4)
DM 1223 26 3.02 (1.34-7.25)
MI
non-DM 6104 188 1.28 (0.95-1.71)
DM 1223 68 1.73 (1.05-2.85)
AHF
non-DM 6104 280 1.07 (0.83-1.37)
DM 1223 100 1.16 (0.76-1.75)
← Favor men
Favor women →
0
0.5
1
5
10

(C)

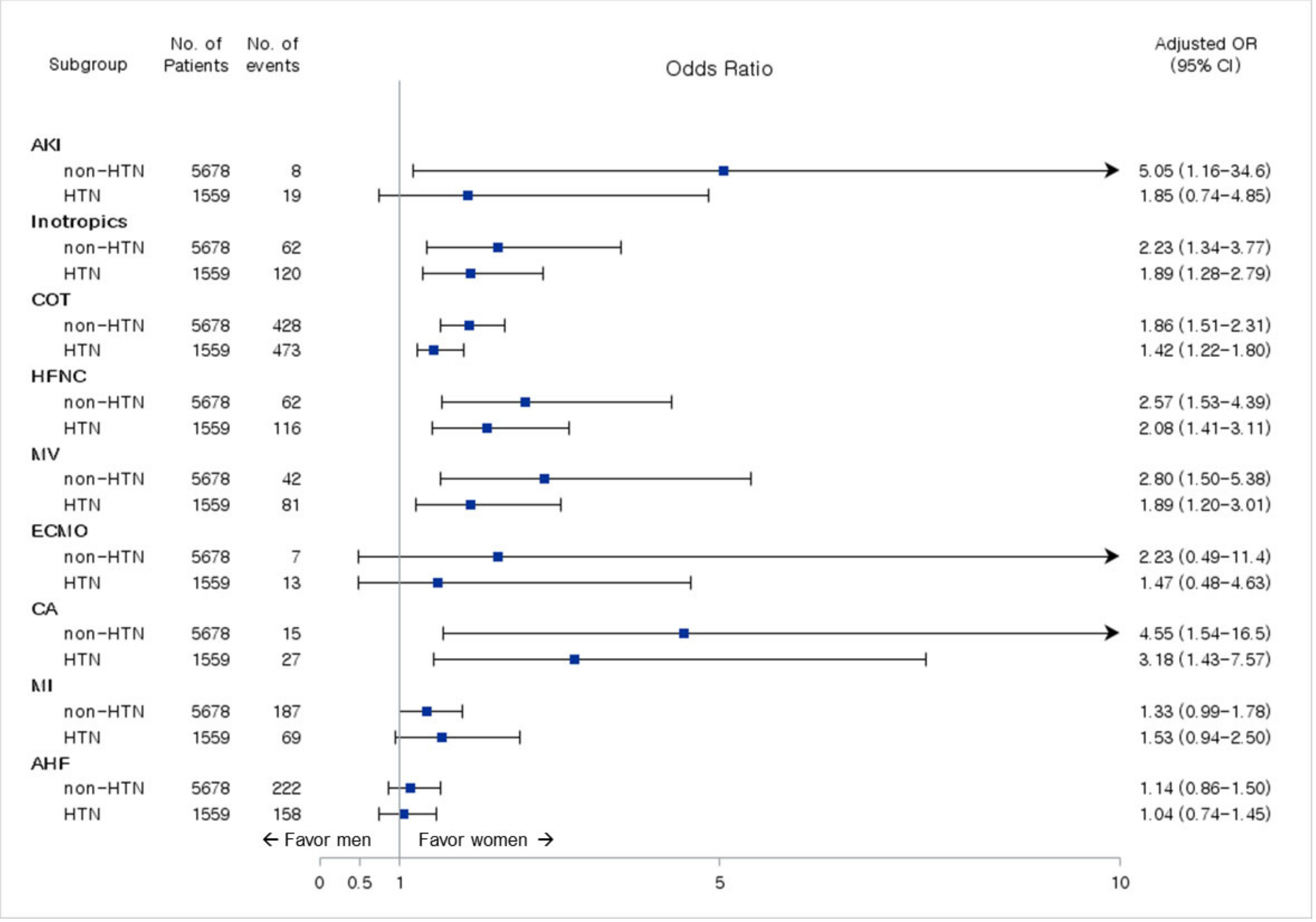

Subgroup
No. of Patients
No. of events
Odds Ratio
Adjusted OR (95% CI)
AKI
non-HTN 5678 8 5.05 (1.16-34.6)
HTN 1559 19 1.85 (0.74-4.85)
Inotropics
non-HTN 5678 62 2.23 (1.34-3.77)
HTN 1559 120 1.89 (1.28-2.79)
COT
non-HTN 5678 428 1.86 (1.51-2.31)
HTN 1559 473 1.42 (1.22-1.80)
HFNC
non-HTN 5678 62 2.57 (1.53-4.39)
HTN 1559 116 2.08 (1.41-3.11)
MV
non-HTN 5678 42 2.80 (1.50-5.38)
HTN 1559 81 1.89 (1.20-3.01)
ECMO
non-HTN 5678 7 2.23 (0.49-11.4)
HTN 1559 13 1.47 (0.48-4.63)
CA
non-HTN 5678 15 4.55 (1.54-16.5)
HTN 1559 27 3.18 (1.43-7.57)
MI
non-HTN 5678 187 1.33 (0.99-1.78)
HTN 1559 69 1.53 (0.94-2.50)
AHF
non-HTN 5678 222 1.14 (0.86-1.50)
HTN 1559 158 1.04 (0.74-1.45)
← Favor men
Favor women →
0
0.5
1
5
10

Figure S3. Forest plot of the association between sex and clinical outcomes in patients according to age (A), DM (B), or HTN (C). Multivariate analyses for the age or DM subgroups were adjusted for age, sex, CCI score, and HTN. Multivariate analyses for the HTN subgroups were adjusted for age, sex, and CCI score.

Abbreviations: OR, odds ratio; CI, confidence interval; DM, diabetes mellitus; HTN, hypertension; AKI, acute kidney injury; COT, conventional oxygen therapy; HFNC, high flow nasal cannula; MV, mechanical ventilation; ECMO, extracorporeal membrane oxygenation; CA, cardiac arrest; MI, myocardial infarction; AHF, acute heart failure; CCI, Charlson comorbidity index

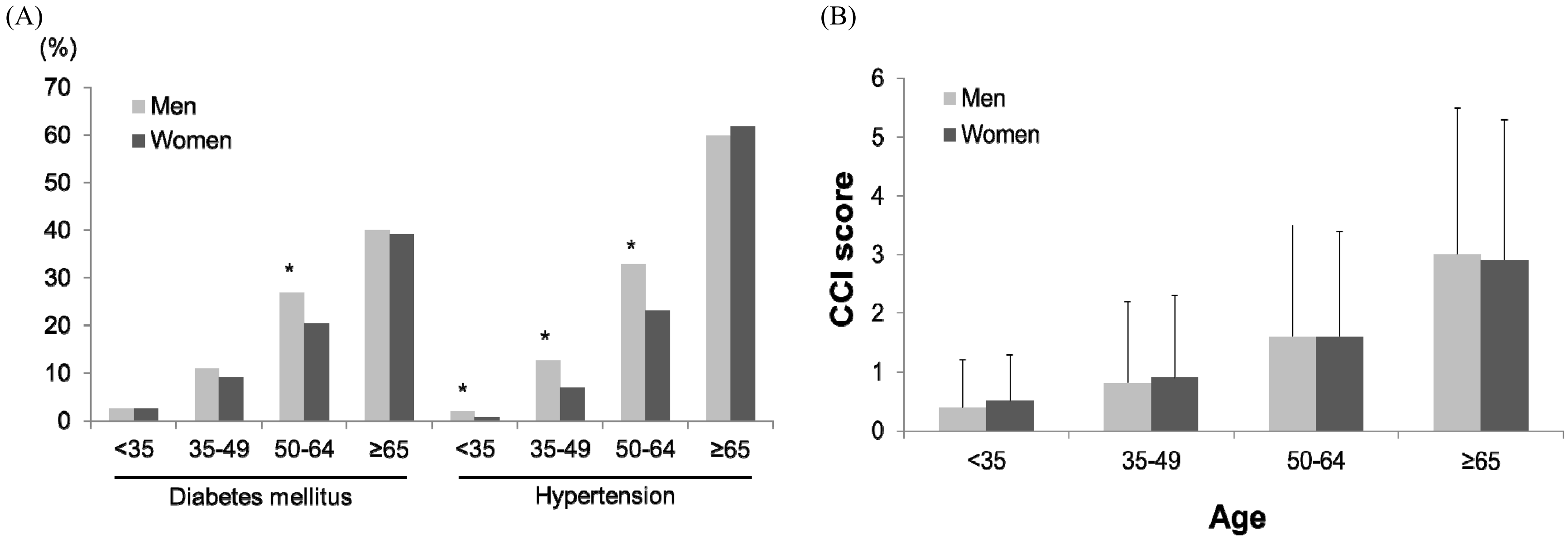

Figure S4. (A) Proportions of patients with diabetes mellitus or hypertension according to sex and age. (B) CCI scores according to sex and age. There were 29 (2.6%) and 32 (2.5%) patients with diabetes mellitus among men and women aged <35 years, 53 (10.9%) and 83 (9.1%) patients among men and women aged 35–49 years, 213 (26.9%) and 276 (20.4%) patients among men and women aged 50–64 years, and 220 (39.9%) and 317 (39.1%) patients among men and women aged ≥65 years, respectively. There were 22 (1.9%) and 9 (0.7%) patients with hypertension among men and women aged <35 years, 61 (12.6%) and 63 (6.9%) patients among men and women aged 35–49 years, 260 (32.8%) and 313 (23.1%) patients among men and women aged 50–64 years, and 330 (59.8%) and 501 (61.8%) patients among men and women aged ≥65 years, respectively. *$P < 0.05$ vs. women in same age group.

Abbreviations: CCI, Charlson comorbidity index
